# The use of silver nanoparticles in pigs – An invited review

**DOI:** 10.17221/101/2024-VETMED

**Published:** 2025-03-24

**Authors:** Nikola Hodkovicova, Miroslav Machacek, Jana Cahova, Jerico Consolacion, Andrzej Siwicki, Zygmunt Pejsak, Martin Svoboda

**Affiliations:** ^1^Department of Infectious Diseases and Preventive Medicine, Veterinary Research Institute, Brno, Czech Republic; ^2^Department of Animal Protection and Welfare & Veterinary Public Health, Faculty of Veterinary Hygiene and Ecology, University of Veterinary Sciences Brno, Brno, Czech Republic; ^3^Department of Agricultural Sciences, College of Agriculture, Forestry, and Environmental Sciences, Mindanao State University at Naawan, Naawan, Philippines; ^4^Department of Animal Science and Food Processing, Faculty of Tropical Agrisciences, Czech University of Life Sciences Prague, Prague, Czech Republic; ^5^Proteon Pharmaceuticals S.A., Lodz, Poland; ^6^Faculty of Veterinary Medicine, Agriculture University, Krakow, Poland; ^7^Ruminant and Swine Clinic, University of Veterinary Sciences Brno, Brno, Czech Republic

**Keywords:** antibiotics, antimicrobial, disinfectants, nanotechnology, swine, toxicity

## Abstract

Silver nanoparticles (AgNPs) have attracted significant interest in veterinary medicine due to their unique properties, including enhanced stability, greater antimicrobial efficacy, and reduced toxicity compared to traditional silver salts. Their applications span various areas of veterinary practice, such as dermatology, wound management, infection prevention, drug delivery, and disinfection. This review explores their use in pigs, highlighting their role as feed additives to prevent diarrhoea, as antibacterial agents in semen extenders, and veterinary dermatology. AgNPs possess broad-spectrum antibacterial activity against both Gram-positive and Gram-negative bacteria, positioning them as a promising alternative to antibiotics in addressing antibiotic resistance. Additionally, AgNPs have shown antiviral potential, though the exact mechanism of action remains unclear. The review examines the antibacterial and antiviral properties of AgNPs, their utility in facility sanitation, and their potential toxicity to pigs. While AgNPs offer significant benefits in veterinary applications, concerns about their toxicity persist. Efforts to reduce this toxicity, such as surface modifications or combining AgNPs with other substances, are under investigation. Further research is essential to fully understand the potential applications and safety of AgNPs in pig medicine.

## INTRODUCTION

In recent years, nanotechnology has experienced a huge increase in use in various industries, including veterinary medicine and animal husbandry in general. Nanomaterials are used as part of medicines, vaccines, or as feed additives in various forms (Youssef et al. 2019; Danchuk et al. 2023). Nanoparticles in general are particles in size < 100 nm with enormous surface-to-volume proportion. Due to their small size, they can enter cells, tissues, and organs (Youssef et al. 2019; Gelaye 2023). Thus, nanoparticles were studied for their potential to act as carriers for drug delivery applications as they can carry various medications inside or on their surface. Moreover, they can be synthetised relatively easily at various sizes, and shapes and their surface can be modified with different moieties (Youssef et al. 2019; Pasparakis 2022).

Nanoparticles can be divided according to many criteria, including their structure, origin, shapes, or way of use. In this review, we take a closer look at silver nanoparticles (AgNPs), which we classify as metallic nanoparticles according to their structure. Metallic particles are formed by metallic centres coated by a protected layer with or without various loadings on the surface (Youssef et al. 2019) and, especially the silver and gold ones are commonly used in the field of medicine (Pasparakis 2022).

Regarding veterinary medicine, the AgNPs are mostly valued for their high stability, lower dose required, greater antimicrobial activity and lower toxicity for eukaryotes (Abad-Alvaro et al. 2019). Given that AgNPs have several applications in the veterinary field, including dermatology and wound care as they help in controlling infections, function as drug deliverers, and as part of disinfectants help sanitise various equipment, housing and environment (Danchuk et al. 2023; Gelaye 2023).

AgNPs are well-known for their antibacterial, antiviral, and antifungal properties, though the precise mechanisms behind these effects remain only partially understood. It is believed that their toxicity towards microbes arises from their ability to bind directly to cell membranes, exerting a biocidal effect that triggers the production of reactive oxygen species (ROS) and free radicals. These reactive molecules then damage intracellular organelles, leading to apoptosis. Other proposed mechanisms include the adhesion of AgNPs to microorganisms, allowing the particles to infiltrate the cells, causing membrane damage that results in cell content leakage and lysis, destruction, or killing. Additionally, AgNPs are thought to have the capacity to alter DNA and disrupt protein functions (Ferdous and Nemmar 2020; Egbuna et al. 2021; Luceri et al. 2023). Moreover, AgNPs also exhibit anticancer properties (Egbuna et al. 2021).

AgNPs are effective against a broad range of bacteria, including both Gram-positive (e.g. *Aci-netobacter, Escherichia, Pseudomonas, Salmonella, Vibrio*) and Gram-negative strains (e.g. *Bacillus, Clostridium, Enterococcus, Listeria, Staphylococcus, Streptococcus*) (Banach et al. 2016; Salomoni et al. 2017). This makes them a promising tool in medicine for combating antibiotic resistance (Pasparakis 2022; Danchuk et al. 2023). The antibacterial activity of AgNPs is primarily due to their ability to release Ag+ ions, which have a strong affinity for organic compounds containing sulphur and phosphorus. By interacting with thiol groups, these ions disrupt the structural integrity of organic molecules (Ferdous and Nemmar 2020; Luceri et al. 2023). Smaller AgNPs, with their larger surface area, release Ag+ ions at a higher rate into the surrounding environment. This process hinders DNA replication and impairs the function of respiratory enzymes, the mitochondrial respiratory chain, and ATP synthesis. Furthermore, the released Ag+ ions promote the formation of ROS, leading to cell damage and potentially triggering apoptosis (Bruna et al. 2021; Pasparakis 2022; Luceri et al. 2023).

The number of studies focusing on the antiviral activity of AgNPs in animals is limited and the provable effects need more extensive studies. Regarding pigs, its efficacy against African swine fever virus was proved as the contamination of pigsties was significantly reduced if sprayed with a solution of AgNPs at the concentration of 25 ppm (Dung et al. 2020). The study confirmed that AgNPs have strong antiviral potential in transferring of this disease which has no treatment or vaccination to this day. Next, the potential efficacy of AgNPs against the Porcine Reproductive and Respiratory Syndrome Virus (PRRSV), a highly contagious virus that causes severe fever and diarrhoea in pigs, was investigated by Ren et al. (2023). In this study, rhein, an active ingredient known from traditional Chinese medicine, was combined with silver nanocomposites. The results demonstrated that the antiviral efficacy of rhein/AgNPs against PRRSV is influenced by the particles’ morphology. The study highlighted the potential of rhein/AgNPs to reduce the production of ROS.

Due to the mechanism of action, which is the release of Ag+ ions, not only antimicrobial efficacy was proven, but also systemic toxicity, which correlates precisely with the amount of Ag+ released. The existing strategies to mitigate this toxicity are based on modifying the surface of the particles or combining them with other substances (Pasparakis 2022).

It is important to mention that nanoparticles can be produced, characterised, utilised, etc. in many ways, and overall, nanotechnology is an enormously vast subject that is constantly developing. However, during our research, we found a lack of information on the issue of the use of nanotechnology in veterinary medicine, specifically in pigs. The possibilities of using AgNPs in swine medicine has therefore become the main subject of this review. The study discusses in more detail the following areas of AgNP usage in pigs – as feed additives to prevent diarrhoea, as an antibacterial agent in semen extenders and as an important tool in veterinary dermatology. Also, the antibacterial and antiviral potential of AgNPs were discussed including the potential for sanitation of internal and external facilities. Last but not least, the toxic potential of AgNPs for pigs is also included.

## THE USE OF SILVER AND SILVER NANOPARTICLES

Historically, silver has been known to have healing properties and was used in animal treatment, however, it could not compete with cheaper antibiotics then. Silver was traditionally used in the form of salts; in particular, silver nitrates (Zyro et al. 2023). Silver in this form is converted to the less effective silver chloride in the stomach and bloodstream that can, moreover, form insoluble complexes. Also, silver nitrate is unstable and can be toxic to tissues (Atiyeh et al. 2007; Fondevila 2010).

Currently, the easier and cheaper production of AgNPs using new technologies means a better perspective for the introduction of the use of AgNPs in veterinary practice and to fully explore its healing potential (Bakowski et al. 2018). Compared to conventional silver salts, AgNPs showed better stability and antimicrobial effects, are more resistant to deactivation by gastric acids and have a lower absorption through the intestinal mucosa, thus reducing the risk of their toxicity. Overall, AgNPs show less toxicity to eukaryotic cells (Fondevila et al. 2009; Dakal et al. 2016; Zyro et al. 2023).

Generally, AgNPs are particles of less than 100 nanometres in size and it has been proven that AgNPs show positive effects on the production parameters of animals already in very low concentrations (Abdelsalam et al. 2019). This is particularly important compared to traditionally used elements such as zinc and copper, which have been used in concentrations that are ten to hundred times greater compared to AgNPs; the selection of AgNPs is, therefore, also important for reducing the environmental burden (Fondevila 2010). However, an increase in the applied dose of AgNPs led to higher retention of AgNPs in the liver of pigs (Fondevila et al. 2009) and in other tissues across different species (Noga et al. 2023).

The AgNPs are utilised mainly for their strong antibacterial effects. The bactericidal effects of AgNPs are related to their larger surface area for interaction with the bacterial surface (Bakowski et al. 2018; Ahmad et al. 2020). The final effects of AgNPs depend mainly on their size and concentration, on the coating substances and the substrate (Bakowski et al. 2018). However, the exact mechanism of activity has not been completely explained as yet. One of the theories is that AgNPs can release silver ions (Ag+), that can interact with nucleic acids and disrupt bacterial cell walls through electrostatic attraction. This interaction increases cell permeability, deactivates respiratory enzymes, produces reactive oxygen species (ROS), and interferes with ATP production (Ahmad et al. 2020).

Overall, the AgNPs show antibacterial activity against various Gram-positive and Gram-negative bacteria (Bruna et al. 2021). Moreover, current studies describe the improvement of the antibacterial effects of AgNPs using various coatings substances, mostly antibiotics (Bruna et al. 2021).

For example, Muenraya et al. (2022) found that AgNPs conjugated with colistin (Col-AgNPs) have higher *in vitro* activity against Gram-negative bacteria (*E. coli*, *K. pneumoniae* and *P. aeruginosa*) compared to AgNPs and colistin alone. In another study by Sirin et al. (2023), nanostructured antimicrobials were obtained by conjugating colistin (COL) and meropenem (MEM) antibiotics with biosynthesised AgNPs. It was *in vitro* tested against multidrug-resistant (MDR) Gram-negative bacteria *E. coli* and *K. pneumoniae*. Antibiotic-conjugated nanoparticles exhibited better antimicrobial activity and lower MIC values on the tested bacterial strains than antibiotics alone. The results suggest that antibiotic-conjugated nanoparticles have the potential to be used as effective antimicrobial products against MDR *E. coli* and *K. pneumoniae* strains.

## THE USE OF AgNPs AS DISINFECTANTS

Disinfectants are substances used against microbes, intending to secure the health of humans and animals. Disinfectants are widely used across various industries, including hospitals, the food industry, and veterinary care. Nowadays, an increasing number of microorganisms are becoming resistant to antibiotics and disinfectants. For this reason, new types of disinfectants are being developed that do not lead to bacterial resistance. One approach to creating new disinfectants is by utilising nanotechnology-based solutions (Edwards-Jones 2009).

The unique properties of nanomaterials enable their multifunctional use as disinfectants by optimising their physicochemical characteristics. Nanomaterials can be applied in food preservation, water disinfection, and the sanitisation of devices and surfaces (Gupta and Silver 1998). Furthermore, nanomaterials have demonstrated effectiveness against a variety of bacterial pathogens. As a result, they are increasingly being used as disinfectants (Vargas-Reus et al. 2012; Yang et al. 2021).

Various substances can be used as nanomaterial-based disinfectants. Researchers have identified several metallic nanomaterials with disinfectant properties, including gold (AuNPs) and gold oxide (Au_2_O_3_NPs), silver (AgNPs), copper oxide (CuONPs), magnesium oxide (MgONPs), titanium dioxide (TiO_2_NPs), zinc oxide (ZnONPs), iron oxide (Fe_2_O_3_NPs; Fe_3_O_4_NPs), platinum (PtNPs), and selenium nanoparticles (SeNPs) (Ruparelia et al. 2008; Velayutham et al. 2012; Kojouri et al. 2012; Noori et al. 2014; Hassan et al. 2015; Elemike et al. 2017; Hamdy et al. 2018; Kalinska et al. 2019). The effectiveness of nanoparticle-based disinfectants is attributed to the enlarged surface area of the active substance. The disinfectant effect is enhanced by increasing the surface area, even with the same dosage. The size and shape of particles play a crucial role in maximising their disinfection efficacy (Pantic 2013).

For example, copper nanoparticles (CuNPs) and copper oxide nanoparticles (CuONPs) have demonstrated a significant reduction in virus levels (> 99.99%) at a 1.0% (w/v) concentration against PRRSV. CuNPs, in particular, were the most effective even at lower concentrations (Graham et al. 2021). However, the disinfectants based on AgNPs are currently among the most widely used us they offer the advantage of lower toxicity compared to other silver disinfectants (Pejsak and Tarasiuk 2021).

In general, silver has been used as a disinfectant for centuries. For example, silver was utilised in China for its disinfectant properties over 1 000 years B.C. (Gupta and Silver; 1998; Edwards-Jones; 2009). Today, silver is commonly used in compounds such as silver nitrate (Pantic 2013). The incorporation of nanotechnology is a modern approach to harnessing silver’s disinfectant properties, allowing for the development of disinfectants that do not promote resistance (Edwards-Jones 2009).

The AgNPs with diameters < 10 nm are particularly effective, as they can denature proteins and inhibit DNA replication in microorganisms (Elechiguerra et al. 2005; Wolska et al. 2017). They are utilised for their disinfectant potential across a variety of industries, including air and water filtration, the textile sector, biomedicine, food packaging, and animal husbandry (Deshmukh et al. 2019). Also, they are now commonly used for water and air filtration, as well as in the treatment of skin wounds as they exhibit a broad spectrum of antimicrobial and anti-inflammatory properties (Shi et al. 2019; Chen et al. 2020). Additionally, the versatility of silver nanoparticles allows them to be combined with specific antibiotics (Czaplewski et al. 2016; Chojniak et al. 2018).

AgNPs are utilised in animal breeding facilities due to their antimicrobial, antifungal, and deodorizing properties (Chmielowiec-Korzeniowska et al. 2015). Their use in biosecurity is increasing for both periodic disinfection (when animals are not present) and ongoing disinfection (when animals are present) (Deshmukh et al. 2019). They are applied for surface, water, and tool disinfection, as well as for preventing pathogen transmission. The benefits of using AgNPs as disinfectants include rapid disinfection, effective deodorisation, and low toxicity (Nia 2007).

For example, their high disinfecting effect has been proven in the study by Humberto et al. (2009). The AgNPs demonstrated a rapid disinfection effect against both Gram-positive and Gram-negative bacteria, which is attributed to their high effectiveness. Although the nanoparticles showed more bacteriostatic than bactericidal effects on bacteria spread on surfaces, their impact was still significant.

Regarding pigs, Yang et al. (2021) investigated the antimicrobial effects of AgNPs on wounds in pigs and found them to be effective against *Pseudomonas aeruginosa*, *E. coli*, and *S. aureus*, with no adverse effects observed (Yang et al. 2021). AgNPs also exhibit antimicrobial activity against the African Swine Fever Virus (ASFV) at a concentration of 25 ppm. When applied by spraying, a concentration of 0.78 ppm was found to be non-toxic to porcine alveolar cells (Dung et al. 2020). The use of nanotechnologies is also considered in disinfection of animal sperm. For example, Perez-Duran et al. (2020) reported that AgNPs effectively reduced bacterial and fungal growth in swine sperm without altering the bioavailability.

Next, the study by Tarasiuk and Wojciechowski (2021) investigates the effects of implementing weekly disinfection with a preparation containing AgNPs in the presence of animals on reducing the infectious potential in a large-scale swine farm. The research was conducted across three successive groups of sows, their offspring, piglets, and porkers.

The findings demonstrated that AgNP-based disinfection significantly decreased the number of microorganisms contaminating the breeding environment, outperforming the previous practice of periodic disinfection with an iodophor preparation. This reduction in infectious potential was evident across different production stages, leading to improved production outcomes. Specifically, the mortality rate of pigs in rooms disinfected with AgNPs was notably lower than in the positive control group, where iodophor disinfection was used. The study showed additionally that treatment costs in the fattening house were reduced. In the positive control group, the veterinary care cost per individual (from 30 kg to the end of fattening) was 5.69 PLN, whereas, in the experimental group, it was 4.02 PLN, indicating a 30% reduction in treatment costs due to the use of ongoing AgNP disinfection.

## THE POTENTIAL USE OF NANO-PARTICLES AS FEED ADDITIVES

Animal nutrition plays a crucial role in agriculture, significantly affecting animal health, growth, and productivity, as well as consumer safety and product quality. Feed additives enhance the quality and flavour of animal diets and help protect them against environmental stressors by improving nutrient efficiency (Dumlu 2024). Traditionally, growth promoters, were used as feed additives, often relying heavily on antibiotics, However, modern agriculture has shifted towards natural feed additives to produce safer products and reduce antibiotic resistance and environmental impact (Ozdemir et al. 2022). Nanoparticles as feed additives present a promising solution due to their high surface reactivity and bioavailability, enabling good results at lower doses. These nanoparticle supplements can easily penetrate the gastrointestinal barrier and enter cells more rapidly, leading to higher absorption rates than larger particles. In summary, the use of nanoparticles as feed additives has been demonstrated to improve growth, digestion, and nutrient conversion (Dumlu 2024).

There is a potential of AgNPs to be applied as feed additives also to prevent diarrhoea in pigs. In fact, the period of weaning is one of the most stressful events in a pig’s life. After weaning, there are changes in the digestive system, immune system and behaviour of pigs (Campbell et al. 2013) that lead to an increased risk of diarrhoea in weaned piglets. Improvements in housing, nutrition and breeding management are used to minimise the adverse effects of post-weaning stress (Hodkovicova et al. 2023). Recently, various feed additives have been considered to support the health status of piglets after weaning, however, their application is connected to various problems.

For example, the addition of zinc in the form of zinc oxide (ZnO) in high, therapeutic doses to feed (2 000–4 000 mg of ZnO per kg of feed) was often used to prevent diarrhoea in piglets after weaning (Hansen et al. 2022). This effect can be explained by the fact that zinc medication has a positive effect on maintaining the stability of the intestinal microflora, on the diversity of coliform bacteria and is important for growth and immune functions (Katouli et al. 1999). The inhibitory effect of ZnO on the growth of some bacteria (e.g., *Staphylococcus aureus*, *Escherichia coli*) was also found *in vitro* (Sawai 2003; Hansen et al. 2022). However, pigs can absorb only up to 25% of ZnO in their diet, which may lead to environmental pollution through faeces (Bai et al. 2019). Based on that, since June 2022, zinc may only be used as an additive in an amount that meets its daily requirement for pigs. The permitted maximum concentration of zinc in feed for pigs, i.e. 150 mg/kg, is stated in the Commission Implementing Regulation (EU) 2016/1095 (European Commision 2016).

Another option to reduce the effects of stress in weaned piglets and increase weight gain is to add copper to the feed (Bikker et al. 2016). Because copper can alter the development and composition of bacterial populations in the intestine, its positive effects are given by its bacteriostatic and bactericidal effects properties (Hojberg et al. 2005; Galiotto Miranda et al. 2024). Recent studies have shown the positive effect of copper sulphate at a dose of 100 mg to 250 mg per kg by promoting the feed intake, the growth of piglets, and the development of their intestines after weaning (Espinosa and Stein 2021). However, the use of high doses of copper sulphate in pig feed has resulted in antagonism with other dietary components and also raises environmental concerns due to high concentrations of copper excreted in faeces (Zhao et al. 2014). Due to the potentially negative effects on the environment, the EU has adopted measures that reduce the maximum permitted concentration of copper in feed by using a total maximum copper concentration of 150 mg per kg of complete feed for suckling piglets and for piglets up to 4 weeks after weaning (EU Regulation 2018/1039) (European Commision 2018).

Both of the mentioned EU Regulations eventually led to the need to look for alternatives. The addition of metal-containing nanoparticles to pig feed stands for a promising alternative solution as their advantage is the possibility of using very low doses while maintaining their effectiveness (Michalak et al. 2022). In recent years, there has been a thrive in the use of silver nanoparticles (AgNPs) as a feed additive in practical terms, especially for the period after piglets are weaned (Danchuk et al. 2023). In this particular review we will further focus specifically on silver nanoparticles and their usage in the pig farms.

Currently, the development of new industrial technologies enabling easier and cheaper production of AgNPs leads to considerations of their use as feed additives. However, there are still very little data available in the literature regarding the use of AgNPs as a feed additive in animals. In general, it can be stated that more attention has been paid to the use of AgNPs as a feed additive in poultry. Studies by Al-Sultan et al. (2022) and Hassanen et al. (2023) demonstrated that AgNPs, especially when chitosan-coated, can improve growth performance in broiler chickens by enhancing body weight and feed conversion ratio. An increase in AgNP dose significantly reduced caecal lactose-positive and enterococci bacteria populations while numerically increasing lactobacilli counts. However, higher doses of uncoated AgNPs caused dose-dependent tissue lesions and silver residues in meat, while chitosan-coated AgNPs at 0.5 ppm were shown to be both effective and safe. Available research on poultry indicates that exceeding a certain dose of AgNPs can lead to negative effects, such as histological lesions in organs and elevated silver concentrations in muscle tissue. However, similar data for pigs are limited.

The use of AgNPs as feed additives in pigs was studied by Fondevila et al. (2009) who tested the effectiveness of AgNPs *in vitro* and *in vivo*. During the *in vitro* experiment, the concentration of AgNPs was increased from 0 to 25, 50 or 100 ppm. The proportion of coliform bacteria in pig’s ileum contents was linearly reduced while no effect was observed on lactobacilli proportion. It follows that the count of bacteria with positive probiotic effects outweighed the count of potentially pathogenic bacteria. Regarding the *in vivo* experiment, weaned piglets were subjected to the effects of AgNPs in their diet starting with weaning and lasting for the following 35 days, i.e. weight of 5 kg to 20 kg. In the AgNPs at doses of 20 and 40 ppm, a significant reduction of coliform bacteria in ileal contents was found. Above that, at a dose of 20 ppm, there was a reduction in the concentration of pathogenic *Clostridium perfringens* and *C. histolyticum*. The study also included a risk assessment for consumers of pork products and, following AgNPs administration, no retention of silver was detected in the kidneys and muscles. However, silver was detected in the liver, with concentrations of 0.44 μg/g at 20 ppm and 0.84 μg/g at 40 ppm meaning that AgNP exposure can potentially pose a risk for the end consumer.

The following studies tried to find whether the use of carriers with nanoparticles can affect their retention in the organism and thus find an ideal ratio between the impact on the consumer and the environment. Recently, the clay material showed promising attributes for AgNP carriers.

In the study of Abad-Alvaro et al. (2019), AgNPs were bound to two types of clay nanocomposites, i.e. sepiolite-Ag and kaolinite-Ag, that were used as AgNP carriers. The aim was to find whether AgNPs bound to these different carriers will differ in the amount of silver released in the digestive tract and whether differences in silver retention in the organism will occur. During the *in vitro* experiment and three-step digestibility assay corresponding to stomach, small, and large intestines simulations, less than 1% of the total silver was released in the stomach. That was explained by the formation of silver chloride on the nanocomposite surface. In the case of the intestine’s simulation, a larger amount of silver was released from kaolinite-Ag compared to sepiolite-Ag, i.e. 17% vs 7%, showing a higher retention rate of silver by sepiolite. In an *in vivo* experiment, two groups of piglets were fed AgNPs bound to similar clay nanocomposites at a dose of 20 mg/kg feed from weaning for 35 days, then switched to normal feed without AgNPs until day 62. On days 14 and 62, several pigs were slaughtered, and their liver, muscle, and rectal content samples were analysed for silver content. Silver concentration in muscle tissue was below detection in all groups, but it was detected in the liver and faeces. No statistically significant differences in silver content were found between groups fed different clay materials as AgNP carriers.

In conclusion, there are still many unanswered questions about using AgNPs as a feed additive in pigs. Key information is lacking on the dosage levels at which negative effects, such as histopathological changes in tissues, begin to appear. Additionally, it is crucial to determine whether higher doses of AgNPs could lead to silver accumulation in tissues, particularly in muscles. Another promising area of research is exploring whether the efficacy of AgNPs can be enhanced by using different coating substances. Currently, no long-term studies have evaluated the effectiveness of adding AgNPs to pig feed as a method to prevent post-weaning diarrhoea in piglets. Future experiments should aim to compare the efficacy of AgNPs with existing methods, such as using zinc or organic acids.

## UTILISATION OF AgNPs AS AN ANTI-BACTERIAL AGENT IN BOAR SEMEN EXTENDERS AND ITS IMPACT ON SPERM QUALITY

Boar semen collection is highly susceptible to bacterial contamination, which can compromise sperm viability and hinder sow conception (Althouse et al. 2008). To combat this, prophylactic antibiotics are commonly added to semen extenders to inhibit bacterial growth and prolong sperm lifespan. However, these extenders can also create an environment conducive to bacterial proliferation (Karageorgiou et al. 2016), and the overuse of antibiotics poses the risk of developing bacterial resistance. Consequently, alternative antibacterial agents, such as nanoparticles made from metals like silver (Rai et al. 2009) or metal oxides like iron oxide (Azam et al. 2012), are being explored as alternatives. Currently, there is limited research on the use of silver nanoparticles (AgNPs) in boar semen extenders, with studies by Basioura et al. (2020) and Perez-Duran et al. (2020) focusing on sperm quality and antimicrobial properties.

Basioura et al. (2020) found that adding AgNPs (Ag/Fe NPs with a 30 nm diameter, containing 5% zero-valent iron at a concentration of 0.128 mg/ml of semen) to boar semen extenders led to significant negative effects on several sperm motion parameters, including total motility, progressive motility, curvilinear velocity, average path velocity, amplitude of lateral head displacement, and beat cross frequency, between 0 and 24 h after treatment. No sperm activity was observed after 48 h of incubation. These sperm kinetics are crucial for determining sperm motility and survival during ejaculation, semen collection, processing, and for use in artificial insemination or cryopreservation. Thus, careful consideration is needed when incorporating AgNPs into extenders, as sperm cells are highly sensitive and metabolically dependent.

Perez-Duran et al. (2020) assessed the effectiveness of AgNPs (10–20 nm) in inhibiting the growth of *Staphylococcus aureus* and their potential impact on sperm. The *S. aureus* growth decreased with AgNP concentrations ranging from 0.4 mM to 10 mM after 22 h of incubation at 37 °C. Regarding sperm viability, AgNP treatment up to 10 mM had no adverse effects, as assessed by mitochondrial metabolism and membrane integrity. Sperm morphology analysis revealed that AgNP concentrations up to 4 mM were harmless after 1 h of incubation at room temperature (25–27 °C). While the study suggests that AgNPs could be an alternative to antibiotics in semen extenders, its limitation lies in evaluating the sperm effects after only a short incubation period. In practice, AgNPs would be in contact with sperm for much longer.

Initially, AgNPs were seen as a potential alternative to antibiotics in semen extenders, but their detrimental effects on certain sperm qualities during short-term incubation must be considered. Further research is necessary to determine the optimal AgNP dosage and assess its long-term effects on sperm quality during extended storage. Additionally, it is important to understand the biochemical pathways involved, as these may influence sperm motility in boar semen, which is critical for commercial pig production.

## THE POTENTIAL APPLICATION OF AgNPs IN PORCINE DERMATOLOGY

Nanotechnology finds its application in various medical practices as nanoparticles are applied in therapeutical, preventive, and diagnostic indications including immunisation and disinfectants. Nanomaterials incorporated into drugs help maintain consistent drug concentrations in the blood and tissues, including the skin. This mechanism enhances therapeutic outcomes, offering exceptional antibacterial and antiviral effects. (Youssef et al. 2019). Dermatotherapies have utilised silver for centuries; however, the limitations of these therapies include skin discolouration, incomplete healing, and disruption of neural and respiratory systems together with higher cost (Nqakala et al. 2021). The metallic nanoparticles possess the ideal properties for wound healing; due to their small size can penetrate deep into the wound and better adhere to the damaged area. Also, due to a high surface area to volume ratio, they serve as a transporter for other drugs and can speed up their effects. They also benefit from high mechanical strength, and anti-inflammatory and antimicrobial potential (Nqakala et al. 2021; Danchuk et al. 2023).

In veterinary medicine, treating most dermatological problems slips incorrectly to antibiotic therapy. However, the overuse of antimicrobial products, which contributes to antimicrobial resistance, poses a significant health risk to both humans and animals. Reducing antibiotic use is essential, and one promising alternative is AgNPs (Shittu et al. 2021). Recent studies suggest that AgNPs have low toxicity and are hypoallergenic (Fedota et al. 2019), making them a viable option for traditional wound healing procedures. Despite their healing potential, the use of AgNPs in veterinary dermatology remains limited (Bai et al. 2018).

The antibacterial properties of AgNPs make them highly valued in veterinary dermatology, with potential applications in various dressings and ointments to enhance wound healing. AgNP-based ointments have shown superior healing properties across different animal species, accelerating skin repair, reducing healing time, and minimising scarring (Khafaga et al. 2018; Kitsyuk and Zvyagintseva 2018; Santos et al. 2019).

Moreover, AgNPs showed a promising antibacterial effect in the cream form if combined with herbal extracts (e.g., *Pelargonium sidoides*, *Sambucus nigra*, and *Hypericum perforatum)*, also. In vitro studies evaluated the cream’s effectiveness against *Escherichia coli*, *Staphylococcus aureus*, and *Candida albicans*. The cream exhibited significant antibacterial activity, with the highest sensitivity against *E. coli*. This was further supported by *in vivo* testing on dogs with various skin diseases, showing successful healing properties and effectiveness of the cream with AgNP content (Popova et al. 2022). Also, a study by Bansod et al. (2015) explored herbal oil combined with biogenic AgNPs in veterinary dermatology. They developed a shampoo, soap, and ointment incorporating these nanoparticles and plant oils. The study found that these products were safe to use and demonstrated significant therapeutic potential with effective disease control, offering a viable alternative therapy for wound healing.

Also, AgNPs have been proven effective against pathogens such as *Staphylococcus aureus*, *Pseu-domonas aeruginosa*, *E. coli*, *Bacillus cereus*, *Listeria innocua*, and *Salmonella choleraesuis* (Pantic 2013). Wounds treated with AgNPs benefit from both hydrophobic and hydrophilic effects; the hydrophobic effect reduces surface contamination, while the hydrophilic effect allows moisture to escape from the wound (Liang et al. 2016).

Regarding pigs, not much information is reported. A study by Nadworny et al. (2010) explored the effectiveness of nanocrystalline silver dressings in the treatment of skin inflammation. The results revealed that the nanocrystalline silver solution exhibited significant anti-inflammatory and pro-healing properties, particularly effective starting at a pH of 9. This suggests that such dressings hold considerable promise for various anti-inflammatory treatments.

The anti-inflammatory potential of AgNPs was already reported; the AgNPs are biocompatible and enable escaping the early inflammatory action that occurs naturally if the foreign particles invade the organism. Due to that, the pro-inflammatory cytokines do not increase, and the immune system is not activated, thus the AgNPs can be used as an anti-inflammatory agent (Nqakala et al. 2021). AgNPs may reduce the expression of pro-inflammatory cytokines, reduce the release of lymphocytes in the wound area and reduce mast cell infiltration to promote better healing without scars (Rajendran et al. 2018).

The most common dermatological problem regarding porcine dermatology is tail biting and lesions arising from it. Biting behaviour is a common welfare issue in commercial pig production systems, leading to significant economic losses. Not only does it cause pain and lead to deformation and abscesses, but tail docking can also trigger an uncontrolled stress response (Morrison and Hemsworth 2020). Tail biting can be classified into aggressive and non-aggressive types. Aggressive biting can be mitigated by enhancing welfare conditions, such as improving early socialisation, addressing undernutrition, and managing cross-fostering (Svoboda et al. 2023). Conversely, non-aggressive biting is often exacerbated by cross-fostering, undernutrition, and competition among individuals. Lesions caused by tail biting are categorised as minor (wounds < 2 cm in diameter or length) or major (≥ 2 cm), based on the size and severity of tissue damage (Valros et al. 2020). Both types of biting typically target the tail or ears, resulting in skin lesions in post-weaning and fattening pigs (Prunier et al. 2020). Affected areas require treatment with antiseptic sprays and broad-spectrum antibiotics to prevent infection. An alternative treatment could involve dressings or ointments containing AgNPs, which offer potential benefits due to their anti-inflammatory and antibacterial properties.

Porowski and Wojciechowski (2023) discuss the use of AgNPs in treating injuries caused by acute cannibalism in pigs, which manifests itself as ear biting, tail biting, and sows biting their offspring. Such injuries often lead to secondary bacterial infections, primarily caused by *Staphylococcus* spp. and *Streptococcus* spp., resulting in widespread skin inflammation, necrosis, and even death. The authors present a compelling case involving the treatment of severe ear damage at high risk of necrosis using AgNPs. A dual-approach therapy was applied: the injured ear was sprayed with an AgNP aerosol preparation three times daily, while ongoing disinfection with AgNPs was conducted three times a week to prevent the spread of microorganisms that could infect the injured ear tissues within the piglet population. After 16 days, the wound was fully healed. Moreover, it was noted that the ongoing disinfection of piglet rooms helped to mitigate infectious issues typically associated with the weaning period.

Based on the results of the mentioned studies, the use of AgNPs in veterinary dermatology, especially in wound healing, is promising as part of the antimicrobial resistance policy and could represent a step forward in the new applications of nanotechnology in veterinary medicine. The application of AgNPs could also find its place in the healing of pig wounds, which are often the result of biting and lead to a need for broad-spectrum antibiotic treatment.

## THE TOXICITY OF AgNPs

Nanotechnology is experiencing rapid and tremendous development in its use in various fields of industry. Focused on AgNPs in particular, tons of silver are released into the environment due to industrial waste (Kumar et al. 2022). Therefore, it is necessary to understand and investigate not only the efficacy of AgNPs but also their potential toxicity for living organisms and the environment.

Generally, the free silver ion (Ag^+^) in its aqueous form is responsible for the main toxic effect, such as bluish-grey discolouration of the skin (argyria) or eyes (agryrosis). Moreover, this form of silver can damage liver and kidney tissue, adversely affect the respiratory and intestinal systems, or even cause serious changes in blood cells (Panyala et al. 2008). But if the nanoform of silver is considered, it is important to realise that various properties of nanoparticles are crucial for their final toxicity. The main factors contributing to the toxicity of AgNPs are mainly the following: shape and size, surface coating, and other physico-chemical attributes (e.g., surface area, aspect ratio, crystallinity, agglomeration, etc.), the ability to release free silver ion, administered concentration, route of exposure and exposure time (Abdal Dayem et al. 2017; Ferdous and Nemmar 2020; Egbuna et al. 2021).

The technology of the synthesis process itself is decisive in the final toxicity of nanoparticles. Different types of synthesis produce various sizes and shapes of nanoparticles (spherical, triangular, etc.), which could affect the final toxicity due to their physical properties (Kumar et al. 2022). The shape of nanoparticles influences the cellular uptake mechanism related to their different toxic effects. Generally, spherically shaped particles have lower toxic potential as they interact differently with biological membranes than rod-shaped particles (Ferdous and Nemmar 2020; Egbuna et al. 2021).

An important parameter related to final AgNP toxicity is the size of nanoparticles. In general, smaller size particles are more toxic than larger ones due to a higher surface area-to-volume ratio that increases their reactivity and potential to provoke the formation of reactive oxygen species (ROS) which can cause oxidative stress and damage cells (Abdal Dayem et al. 2017; Egbuna et al. 2021).

Another important step in the production of nanoparticles is the coating, which prevents the aggregation of nanoparticles and stabilises the whole nanostructure but could have an effect on nanoparticle toxicity, also (Akter et al. 2017). The coatings are applied to modify the properties of the final nano molecule by covering the surface with various layers that contribute to the stability and final functionality of the molecule (Egbuna et al. 2021). Certain coatings might be less toxic to uncoated AgNPs, however, the uncoated ones might release more highly toxic free silver ions (Egbuna et al. 2021). For example, Nguyen et al. (2013) claim that uncoated AgNPs suppress inflammatory responses and cause oxidative stress, while coated AgNPs induce toxic effects by cytokine activation. Thus, it is important to consider nanoparticle-coating as a factor affecting the final toxicity.

To the best of our knowledge, no research has yet been conducted on the toxicity of AgNPs in pigs. However, the toxicity of AgNPs was recorded in poultry; for example, the positive impact of AgNPs on growth performance and caecal microbial population diversity was revealed (Al-Sultan et al. 2022). In contrast, the negative effects of AgNPs applied as feed additives were revealed in the form of histological changes in various organs in broilers (Hassanen et al. 2023). However, similar data are not yet available in pigs. Nevertheless, the results presented in poultry might help in organising similar studies in pigs to elucidate the potential toxicological effect of AgNPs to pigs with maximising their positive potential.

Also, several other experiments have demonstrated the cytotoxicity of AgNPs in animals. Based on the studies, the mechanism of toxicity consists in the aggregation of nanoparticles and oxidation of AgNPs to the form of silver oxide, which leads to the release of free silver ions (Ag^+^ and Ag^0^). These ions are able to enter inside the cell by endocytosis, interact with cellular processes including enzyme function and membrane integrity, alter DNA replication and cause mitochondrial dysfunction (Haase et al. 2012; Egbuna et al. 2021). An *in vitro* study revealed impairment of test rat liver cells after AgNPs exposure. The results of this study were represented by the increase of oxidative stress parameters supplemented by the mitochondria damage (Hussain et al. 2005). One of the potential toxic mechanisms of AgNPs consists in the affinity of silver ions for sulphur, and these mechanisms could eventually cause protein and nucleic acid damage leading to cell apoptosis (Haase et al. 2012; Grzelak et al. 2018).

The dosing and the route of AgNPs administration are also decisive for the toxicity evaluation. For example, the dose of AgNPs < 150 μg/kg has a potential liver protection effect, however, the toxic effect rises extensively with increasing concentration and is concentration-dependent (Reshi et al. 2017). Also, the time period of exposure is an important factor as even a low dose of AgNPs (still under the lowest observed adverse effect level concentration) may cause renal inflammation progressing to renal cell necrosis in rats in long-term oral exposure (Tiwari et al. 2017).

Considering the route of administration, the oral is the most common one but is also associated with a potentially increased retention of nanoparticles in the organism. For example, oral toxicity of AgNPs after 13 weeks’ exposure was demonstrated by Kim et al. (2010), where the target organ for nanoparticle accumulation was the liver. Histopathological examination revealed bile duct hyperplasia, fibrosis and pigmentation. Mao et al. (2018) investigated the lethal and sublethal effects of dietary AgNP exposure in *Drosophila melanogaster*. Lethal doses of AgNPs caused delayed development with increasing lethality in young adults. On the other hand, sublethal doses caused a shortened lifespan and greater susceptibility to oxidative stress. In particular, dietary exposure to AgNPs led to excessive production of free radicals and associated induction of oxidative stress responses, DNA damage and intestinal inflammation (Ferdous and Nemmar 2020).

Other routes of administration proved the AgNP toxicity as well. It was reported that inhalation of AgNPs could induce anti-proliferative and apoptotic effects on airway smooth muscle cells in the human body (Ramirez-Lee et al. 2014). Next, intravenous administration to rats led to an increase in the size of the spleen, especially an increase in T and B cell populations accompanied by accumulation of AgNPs in the spleen, liver and lymph nodes. Elevated activities of phosphatase, alanine transaminase, and aspartate transaminase indicated liver damage (De Jong et al. 2013). Absorption of silver particles via skin and its effect on organisms was described in a study by Burd et al. (2007) in monolayer cells, tissue explant, and an animal model. Result of the study shows that some of the commercially available silver-based dressings have a cytotoxic effect that could affect wound healing (e.g. delayed wound healing or inhibition of wound epithelialisation). The cytotoxicity was correlated with silver released from the dressings, which was measured by the concentration of silver in the culture medium.

Due to their generally low toxicity and low effective concentration, AgNPs are widely used, ranging from daily hygiene products to medications and are considered safe for use. However, there is a risk of cumulative toxicity, side effects such as cytotoxicity, oxidative stress, DNA damage, and possible harmful impacts on ecosystems which must be carefully monitored (Tran et al. 2013; Franci et al. 2015).

In conclusion, for a better understanding of the toxicological mechanisms, studies on the toxicological effect of AgNPs on organisms are still needed. Shape, size, structure, and surface chemistry are the critical aspects of toxicological evaluation. Additionally, there is a strong need for long-term studies to assess their toxic potential after prolonged use (Dumlu 2024).

## SUMMARY

Silver nanoparticles (AgNPs) have unique properties, including enhanced stability, antimicrobial efficacy, and reduced toxicity. They already have applications in veterinary medicine, such as dermatology, wound management, infection prevention, and disinfection or as antibacterial agents in semen extenders. Also, AgNPs show promising properties in preventing and treating diarrhoea, suggesting potential future applications as feed additives. Due to their antiviral potential and broad-spectrum antibacterial activity against Gram-positive and Gram-negative bacteria, they offer an alternative to antibiotics.

This review also discusses their utility in facility sanitation. However, given their mechanism of action, they have a potential toxicity to organisms, which is subject of this review, too. Nanotechnology and its application in the field of medicine is a rapidly developing industry that brings incredible possibilities for use in the future.

Thus, further research is needed to fully understand the potential applications and safety of AgNPs in swine medicine.
